# Validation of a Brazilian version of the Checklist for Assessment of Gender Disadvantage (CAGED) in adolescents

**DOI:** 10.1590/1984-0462/2026/44/2025240

**Published:** 2026-08-07

**Authors:** Luciana Lourenção Andreta, Larissa Araújo Galdino da Silva, Matheus Gallo Cruz, Letícia Bernucci de Oliveira, Maria Carolina Garcia Bronharo, Maria Clara Machado Wintruff, Lília D`Souza Li

**Affiliations:** a Centro Universitário Padre Albino, Faculdade de Medicina de Catanduva, Catanduva, SP, Brazil.; b Pontifícia Universidade Católica de Campinas, Campinas, SP, Brazil.; c Universidade Estadual de Campinas, Campinas, SP, Brazil.

**Keywords:** Adolescents, Gender inequity, Validation study., Adolescentes, Desigualdade de gênero, Estudo de validação.

## Abstract

**Objective:**

To validate and culturally adapt a Brazilian version of the Checklist for Assessment of Gender Disadvantage (CAGED) for girls, boys, and gender-diverse adolescents, and determine the optimal cutoff point.

**Methods:**

The original instrument, designed to assess perceptions of gender inequity among girls, was validated alongside a version for boys and gender-diverse people through seven stages: translation, synthesis, back translation, expert evaluation, pre-testing with 29 adolescents, re-evaluation, and clinical validation with 459 adolescents. To assess perceived gender discrimination, in addition to the original binary response options (yes/no), three alternative responses were tested to clarify whether “it never happened” or “it happened but does not bother me”. The Content Validation Index (CVI) was calculated, with scores ≥0.80 indicating acceptable agreement. The scale’s reliability was assessed using Cronbach’s alpha, with a threshold of ≥0.70 indicating good internal consistency. The Self-Reporting Questionnaire (SRQ-20) was used as the gold standard to construct a receiver operating characteristic (ROC) curve and determine the optimal sensitivity, specificity, and cutoff point for the CAGED.

**Results:**

The translation demonstrated excellent agreement, with all CVIs exceeding 0.80. The Cronbach’s alpha in the pre-test was 0.97. Both response formats proved adequate, with CVIs of 0.83 for the binary and 0.79 for the three-response alternatives. The cutoff point with the highest sensitivity (72.08%) and specificity (82.61%) was 4. The area under the ROC curve was 0.823.

**Conclusions:**

The CAGED was successfully translated and culturally adapted for Brazilian adolescents, showing high internal consistency and clarity.

## RESUMO

**Objetivo:**

Validar e adaptar culturalmente a versão brasileira do Checklist for Assessment of Gender Disadvantage (CAGED) para meninas, meninos e adolescentes de gêneros diversos e determinar o melhor ponto de corte.

**Métodos:**

A versão original, destinada a avaliar percepções de desigualdade de gênero entre meninas, foi validada junto com uma versão para meninos e pessoas de gêneros diversos, em sete etapas: tradução, síntese, retrotradução, avaliação por especialistas, pré-teste com 29 adolescentes, reavaliação e validação clínica com 459 adolescentes. Para avaliar a presença de sofrimento por desvantagem de gênero percebida, além das opções de resposta binária original (sim/não), foram testadas três alternativas para esclarecer “se nunca aconteceu” ou “se aconteceu, mas não incomodava”. O Índice de Validação de Conteúdo (IVC) foi aceito se ≥0,80. A confiabilidade foi avaliada pelo alfa de Cronbach, com valores ≥0,70 indicando boa consistência. O Self-Reporting Questionnaire(SRQ-20) foi utilizado como padrão-ouro para construir a curva receiver operating characteristic (ROC) e determinar sensibilidade, especificidade e ponto de corte ideais do CAGED.

**Resultados:**

A tradução apresentou forte concordância, com todos os IVCs acima de 0,80. O alfa de Cronbach no pré-teste foi de 0,97. Ambos os formatos de resposta demonstraram adequação, com IVCs de 0,83 (binária) e 0,79 (três alternativas). O ponto de corte de maior sensibilidade (72,08%) e especificidade (82,61%) foi 4. A área sob a curva ROC foi de 0,823.

**Conclusões:**

A versão brasileira do CAGED foi traduzida e culturalmente adaptada para adolescentes brasileiros, demonstrando alta consistência interna e clara compreensão.

## INTRODUCTION

Gender roles and societal norms are established from birth, shaping social expectations and delineating what is deemed acceptable behavior for individuals within a given culture.^1,2^ These roles may be traditional, characterized by inequality between men and women in familial and professional domains, or egalitarian, emphasizing an equitable division of responsibilities.^3^

Negative gender-related experiences occur when individuals perceive disparities in gender roles and norms, or when their gender identity results in deprivation of opportunities.^4,5^ Such experiences have been associated with adverse mental health outcomes among youth, including increased depressive symptoms,^5^ eating disorders, and suicidal ideation.^4^

Gender-based violence encompasses any form of physical, psychological, sexual, or symbolic assault directed at individuals in vulnerable situations due to their gender identity or sexual orientation.^6^ Prejudice and the subordinate social positioning of women can precipitate violence against them.^7,8^ How gender relations are constructed, along with entrenched cultural beliefs, perpetuate a cycle of victimization, often stigmatizing and silencing survivors.^9^ Symbolic violence, in particular, refers to subtle, often invisible forms of violence exercised through behaviors, ideologies, and institutional practices that naturalize and legitimize inequality.^9^ These mechanisms collectively establish a symbolic framework that transmits and sustains dominant conceptions, often dissimulating the victim’s perception and reinforcing the perpetrator’s power.^10^

According to the World Health Organization (WHO), approximately one in three women will experience physical or sexual violence throughout their lifetime.^7^ The risk is notably higher among young, married women without paid employment^7^ and among women belonging to sexual minorities, who are nearly twice as likely as heterosexual women to be involved in conflicts.^11^ Most incidents of violence occur within domestic and familial settings.^12^ Globally, about 25% of the population believes that a man is justified in hitting his wife, thereby legitimizing such violence and complicating reporting efforts.^13^ Traditional gender roles foster power imbalances that legitimize violence, which over time becomes normalized and trivialized, often resulting in victim-blaming.^12^ Persistent exposure to violence erodes self-esteem and impairs the victims’ capacity to respond or seek help, leading to a sense of resignation and conformity.^12^ Although women are the primary targets, men and people of sexual and gender minorities are also susceptible to violence and coercion.^12^ Therefore, reliable instruments that assess the negative impact of perceived gender inequality are crucial for improving support and intervention strategies for adolescents, particularly girls, who are most affected, as well as boys and people of sexual and gender minorities. The Checklist for Assessment of Gender Disadvantage (CAGED) evaluates the perceptions of gender inequity in women and is associated with the severity of psychological distress.^14^ Despite its relevance, a version of this instrument specifically tailored and validated for the Brazilian sociocultural context remains unavailable.

Thus, the present study aimed to validate and culturally adapt Brazilian versions of the CAGED for girls, boys, and gender-diverse adolescents. In addition, to determine the optimal cutoff point with the highest sensitivity and specificity for the questionnaire, we constructed a receiver operating characteristic (ROC) curve using the Self-Report Questionnaire-20 (SRQ-20) as the gold standard.

## METHOD

This is a cross-sectional observational study of adolescents to validate and culturally adapt a Brazilian version of the CAGED. It was developed in India to assess women’s perceptions of gender inequity.^4^ It contains 15 questions that evaluate four domains: gender discrimination, with three questions, whether male family members had some advantages over women (question 1), whether women were criticized and/or ridiculed (question 10), and whether women’s opinions were valued (question 14); barriers to personal growth, with three questions, whether male family members had more opportunities for study or work (question 2), whether financial difficulties were barriers for women to achieve their goals (question 6), and whether women felt their freedom was diminished (question 15); emotional stress, with three questions, whether women have suffered prejudice for being women and whether they felt anger because of it (question 3), whether they have thought that being dead would be the only solution when dealing with prejudice for being a woman (question 4), and whether they have thought about self-harm (question 5); violence and harassment, with six questions, whether they had been stalked (question 7), whether they felt a lack of support when being stalked (question 8), whether they lacked space and privacy (question 9), whether they had suffered physical violence (question 11), whether they had witnessed domestic violence (question 12), and whether they had been bothered by some pick up lines (question 13).^4^

This work was approved by the local Research Ethics Committee (Certificate of Presentation for Ethical Appreciation — CAAE: 55780622.5.0000.5430 and CAAE: 57821816.80000.5404). The original questionnaire was translated and adapted for use in Brazil, designed for girls to answer, with “yes” or “no” response options for all questions, and enrolled 200 girls. During validation, a second project was proposed to test the modified questionnaire with boys and transgender or non-binary adolescents, with questions 5, 9, 11, 13, 14, and 15 retaining the focus on personal experiences, while the other questions addressed witnessing attitudes towards women. To better characterize negative responses, this version included three response options for questions 1 to 9: “yes, that happens and bothers me”, “that happens, but does not bother me”, and “no, that does not happen”. This adapted version was then applied to another 259 adolescents.

To compare the two response formats (two versus three options), the three-option responses were first analyzed separately. Subsequently, an analysis using two response options (yes and no) was conducted. For this purpose, responses from the three-option scale were recategorized: “yes, that happens and bothers me” was classified as yes, whereas “that happens, but does not bother me” and “no, that does not happen” were grouped as no.

**Figure 1:**
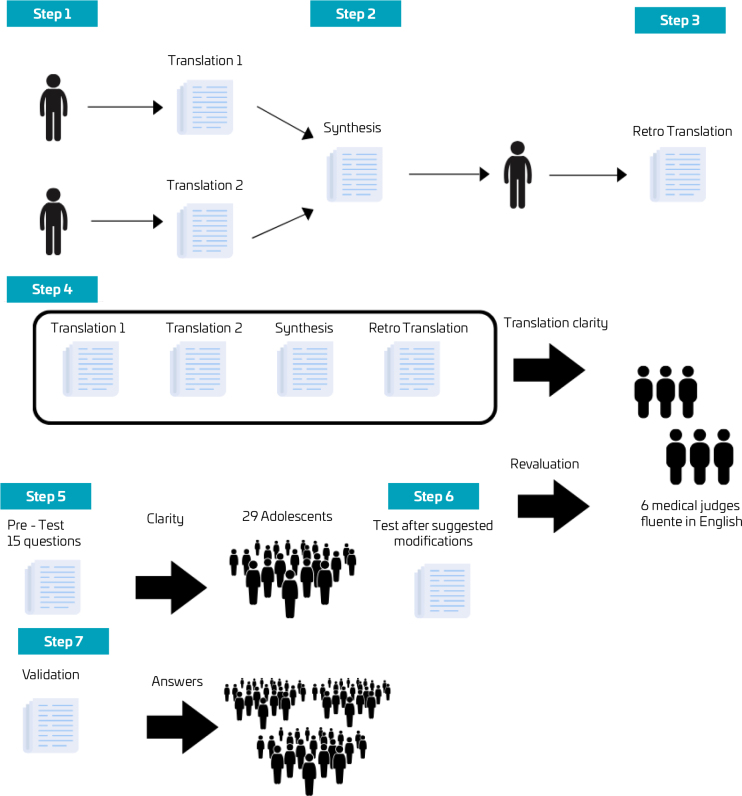
Stages of questionnaire validation of the Checklist for the Assessment of Gender Disadvantage.

Source: Prepared by the authors.

The translation and cultural adaptation were carried out in seven stages: translation, synthesis, back translation, evaluation by a committee of judges, pre-testing, re-evaluation by the committee of judges, and clinical validation^15,16^ (Figure 1).

Stage 1: Translation. Two medical professionals, Brazilian and fluent in English, translated CAGED into Portuguese and provided us with a combined perspective.

Stage 2: Synthesis. The two translations were synthesized into a single translation.

Stage 3: Back-translation. An American citizen who worked as an English teacher while living in Brazil, fluent in Portuguese, back-translated the synthesized version into English.

Stage 4: Evaluation by a Committee of Judges. A committee of six judges, either medical professionals or university professors, fluent in English, evaluated all versions of the questionnaire and answered two questions: to what extent they agreed that the two versions (the original questionnaire and the Portuguese translation) had the same meaning, and how clear they believed the questions were. The response options were Likert-type, with scores ranging from 1 (strongly disagree) to 5 (strongly agree).

The judges reviewed all translations, offered criticisms and suggestions for adjustments to the initial translation, and assisted in decision-making.^17^ They also assessed which response options they thought were more appropriate: the one with two alternatives (“yes” or “no”) or the one with three alternatives (“no, that doesn’t happen”, “that happens and doesn’t bother me”, or “yes, that happens and bothers me”).

Stage 5: Pre-Test. After the judges’ evaluation, 29 adolescents of both sexes and from varying socioeconomic levels assessed the questionnaire. The adolescents were invited from the outpatient adolescent clinics, along with their family members. They were asked if they had difficulties understanding certain words and the meaning of the questions. Responses were on a Likert scale ranging from 1 (“I don’t understand anything”) to 5 (“I understand everything”). They were also asked which response option they considered more appropriate for each question in the questionnaire: the two-alternative or the three-alternative option.

Stage 6: Re-evaluation by the Committee of Judges. All suggestions and criticisms from the judges and adolescents were considered, and the modifications were incorporated into the questionnaire. The judges re-evaluated the questionnaire and answered the questions again: to what extent they agreed that the two versions (the original questionnaire and the translation into Portuguese after the suggested modifications) had the same meaning, and how clear they believed the questions were.

Stage 7: Clinical Validation. Clinical validation was conducted in two cities in São Paulo state. In Catanduva, the municipal education department and the state education board selected the schools; of the 13, six schools (five in highly socially vulnerable areas) were included in the study. In Campinas, the school was chosen by convenience. Of the 2500 students enrolled in these schools, 459 agreed to participate. The inclusion criteria were middle and high school students, both sexes, aged ten to 18. Exclusion criteria included adolescents who could not read or comprehend the questions or who did not sign the assent form. The questionnaire included the question: “What is your gender?” with three response options: boys, girls, and other (transgender, non-binary, etc.).

The questions with the two response options were answered by 200 adolescent girls, and the questions with three response options were answered by 259 adolescents, including 135 boys, 111 girls, and 13 adolescents who identified as other.

The Self-Reporting Questionnaire-20 (SRQ-20) is a brief, self-administered screening tool developed by the WHO to identify common mental disorders, particularly depression and anxiety, in primary healthcare settings, especially within low- and middle-income countries.^18^ The instrument consists of 20 dichotomous (yes/no) items that assess symptoms experienced over the past month, including depressive thoughts, depressive/anxious mood, somatic complaints, and lack of energy. Each item is scored as 0 (no) or 1 (yes), and seven or more “yes” answers are suggestive of some common mental disorder. The SRQ-20 has demonstrated good internal consistency, validity, and reliability across diverse populations, making it a widely accepted screening instrument for mental health research and clinical practice.^19,20^

The SRQ-20 was used as the gold standard in an ROC analysis to assess the sensitivity and specificity of CAGED at each threshold and to determine the optimal cutoff.

Statistical calculations were performed using version 29.0.2.0 (20) of IBM Statistical Package for the Social Sciences (SPSS) Statistics. To calculate the Content Validity Index (CVI), the number of responses with high understanding or agreement scores (understand most of it, understand everything/agreed, and strongly agreed) was divided by the total number of responses to each question. A score ≥ 0.80 was considered acceptable agreement.^21^

Cronbach’s alpha was used to assess the scale’s reliability.^17,18^ A value ≥ 0.7 indicates high reliability; between 0.5 and <0.7 indicates moderate reliability; > 0.2 and 0.5 indicates reasonable reliability; and ≤0.2 indicates low reliability.^22^ The results were considered significant at p<0.05.

## RESULTS

The translation of the questionnaire posed challenges, as some words had multiple translations, altering the original meaning depending on context, e.g., “harassment”. Several modifications were necessary to clarify words that had caused confusion, especially among adolescents. The following words have been changed: in question 1, “male members” for “men (*homens*)”; in question 2, “male members” and “female members” for “men (*homens*)” and “women (*mulheres*)”; in question 3, “discrimination” for “prejudice (*preconceito*)”; in question 4, “ending your life” for “to be dead (*estar morta*)”; in question 7, “harassment or teasing” for “stalking (*perseguir*)”; in question 8, “teased/harassed” for “provoked (*provocação*)” and in question 13, “unwanted sexual advances that have caused you distress” for “pick-up lines that bothered you (*cantadas que te incomodaram*)”.

The pre-test was administered to 29 adolescents with a mean age of 14.14 (standard deviation — SD=2.8), of whom 62% were girls. They made suggestions and assessed the best response options for each question.

Questions 4 and 14 had the lowest CVI (0.73). In question 4, adolescents had difficulty understanding the phrase “to end one’s own life”; in question 14, the word “dislikes” was mainly difficult during the pre-test but not during the clinical validation. All other questions presented a CVI above 0.80 (Table 1).

**Table 1:** Content Validity Index of the responses from the judges and the adolescents’ pre-test for the validation of the Checklist for the Assessment of Gender Disadvantage. In the pre-test (step 5), 29 adolescents answered whether they had difficulty understanding certain words and the meaning of the 15 questions. The scores of the re-evaluation (step 6) of the six judges are shown, indicating how much they agreed that the English and Portuguese versions had the same meaning and how clear the 15 questions were.

Question	CVI (adolescents=29)	CVI (judges=6)	
		Agree	Clarity
1	0.87	1	1
2	0.87	1	1
3	0.87	1	1
4	0.73	0.83	0.83
5	0.87	0.83	0.83
6	0.83	1	1
7	0.90	1	1
8	0.80	1	1
9	0.80	1	1
10	0.87	1	1
11	0.80	1	1
12	0.83	1	1
13	0.83	1	1
14	0.73	1	1
15	0.83	1	1

CVI: Content Validity Index.

Source: Prepared by the authors.

In the judges’ and adolescents’ opinions, both response options were appropriate. After the modifications suggested by the judges and adolescents, the judges re-evaluated the questionnaire, and all CVI scores were above 0.80 (Table 1).

The Cronbach’s alpha coefficient for the pre-test responses was 0.97. The Cronbach’s alpha coefficient for validation-stage responses was 0.83 with two response options and 0.79 with three response options (Table 2).

**Table 2:** Cronbach’s alpha coefficient from the adolescents’ pre-test, the clinical validation with the original questionnaire with two response options, and from the adapted version questionnaire with three response options. The coefficient was calculated from the responses to the Checklist for the Assessment of Gender Disadvantage.

Question	Pre-test, n=29 Cronbach’s alpha=0.971	Validation n=459, 2 response options Cronbach’s alpha=0.827	Validation, n=259, 3 response options. Cronbach’s=0.799
	Cronbach’s alpha if the question is deleted	Cronbach’s alpha if the question is deleted	Cronbach’s alpha if the question is deleted
1	0.970	0.817	0.784
2	0.969	0.816	0.784
3	0.977	0.824	0.788
4	0.970	0.825	0.789
5	0.968	0.822	0.792
6	0.969	0.816	0.797
7	0.970	0.808	0.788
8	0.968	0.818	0.778
9	0.968	0.818	0.781
10	0.967	0.816	0.790
11	0.969	0.814	0.791
12	0.968	0.819	0.797
13	0.968	08.14	0.784
14	0.968	0.813	0.786
15	0.968	0.811	0.781

Source: Prepared by the authors.

Participated in the clinical validation 459 adolescents aged 10–19, with a mean age of 13.9 (SD=2) (Table 3).

**Table 3:** Gender and skin color characteristics of the 459 adolescents participating in the Checklist for the Assessment of Gender Disadvantage clinical validation.

	n (%)
Girls that answered questionnaire with 2 (yes/no) response options	200 (43.6)
Girls that answered questionnaire with 3 response options	111 (24.2)
Boys that answered questionnaire with 3 response options	135 (29.4)
Adolescents who identified as other that answer questionnaire with 3 response options	13 (2.8)
White	212 (46.1)
Brown	180 (39.2)
Black	49 (10.6)
Indigenous	13 (2.9)
Yellow	5 (1.2)

Source: Prepared by the authors.

**Figure 2:**
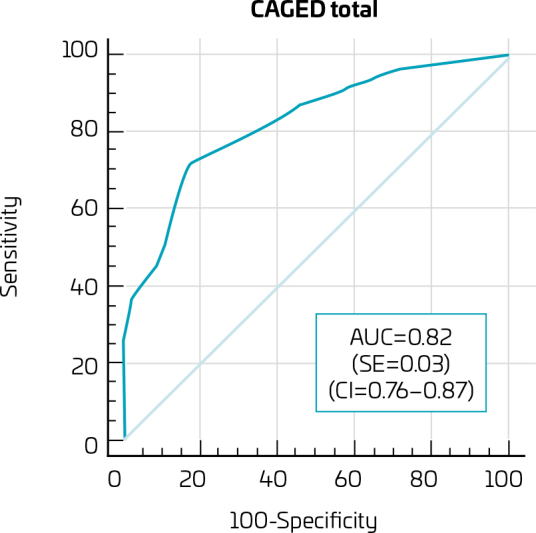
Receiver operating characteristic curve used to determine the optimal cutoff point with the best sensitivity and specificity for the Checklist for the Assessment of Gender Disadvantage, using the Self-Report Questionnaire-20 (SRQ-20) as the gold standard.

Source: Prepared by the authors.

CAGED: Checklist for the Assessment of Gender Disadvantage; AUC: area under the curve; SE: Standard error; CI: confidence interval

The area under the ROC curve was 0.82, and the cutoff point with the highest sensitivity and specificity was 4, yielding 72.08% sensitivity and 82.61% specificity (Figure 2). Considering this cutoff point, 12% of the boys, 46% of people of gender diverse, and 60% of the girls suffered due to gender disadvantage. The three most endorsed gender disadvantages were: stress from gender disadvantage, reported by 63% of adolescent girls, 47% of the boys and 85% of gender-diverse adolescents; thoughts about self-harm, reported by 55% of girls, 23% of boys and 85% of gender-diverse adolescents; and the lack of space and privacy, reported by 50% of girls, 25% of boys, and 53% of gender-diverse adolescents.

## DISCUSSION

A validation and cultural adaptation of the Brazilian version of the CAGED questionnaire was conducted. In addition to translating and adapting the instrument for girls, we also tested and validated a version for boys and gender-diverse adolescents. This process expands the assessment of perceptions of gender inequality to broader groups and facilitates correlations with health and well-being data. The inclusion of an additional negative response option provided greater precision in participants’ perceptions, distinguishing between the absence of discrimination (“it never happened”) and the absence of suffering (“it happens but does not bother me”).

Originally developed in India to assess gender inequality among girls, where disparities in health, education, and nutrition remain pronounced,^4^ the questionnaire required cultural adaptation for use in Brazil. Adapting an existing instrument is more efficient than developing a new one with equivalent measures.^17^ Literal translation proved insufficient to capture cultural nuances; therefore, the adaptation process was essential to avoid misunderstandings and ensure responses were accurate, contextually relevant, and representative of Brazilian adolescents. This step strengthened data validity, as the questions reflected local realities and deepened understanding of the everyday gender inequalities individuals experience. Moreover, the Portuguese version allows cross-cultural comparisons among Portuguese-speaking countries, enhancing its potential impact on research and public policy addressing gender disparities. Despite translation challenges, the results demonstrated good internal consistency, with satisfactory Cronbach’s alphas and content validity indices in all stages, pre-test, judges’ review, and validation, confirming the instrument’s reliability.

Gender norms influence multiple dimensions of life, including well-being, nutrition, hygiene, and health.^23^ Women in leadership positions frequently encounter unequal treatment and biased judgments,^13^ and being female remains associated with poverty and educational and economic disadvantage.^23^ In India, Satyanarayana et al. reported high suffering due to gender inequality across domains: 71.3% of adolescent girls experienced stress from gender disadvantage, 65.7% faced financial obstacles to achieving goals, 59% reported ridicule for being female, and 7.9% had suffered sexual harassment.^4^ In Brazil, the patterns of disadvantage differed, and a higher proportion of sexual harassment was observed. Alarming national data from 2024 revealed that four women were victims of femicide daily, with increases of 0.7% in femicides, 19% in attempted femicides, and 87,545 registered cases of rape, the highest number since the beginning of historical records.^24^

These figures have remained virtually unchanged over the past decade, highlighting the persistence of gender-related murders and violence against women and gender minorities.^23^ Social norms and local culture often normalize such violence. Thus, translating and validating an instrument capable of measuring gender disadvantages represents a crucial step toward promoting discussion and developing interventions to challenge and transform this reality.

Traditional gender expectations also negatively affect men, who may engage in risky behaviors, such as substance use and unprotected sex, to conform to ideals of strength and toughness.^22^ Ermis-Mert, analyzing World Values Survey data, found that greater gender equality correlates with higher happiness and life satisfaction among both men and women.^25^

Using the SRQ-20 as the gold standard enabled us to establish the optimal cutoff for the CAGED, thereby improving the identification of individuals experiencing distress related to gender disadvantage.

Some limitations must be acknowledged: the clinical validation relied on a convenience sample of two cities in the countryside of São Paulo that volunteered, which may introduce selection bias and limit generalizability to other regions of the country. Additionally, as the questionnaire was administered in schools, it may not represent the perceptions of the broader adolescent population.

In conclusion, the translation and cultural adaptation of the CAGED into Brazilian Portuguese demonstrated high internal consistency and good comprehension among adolescents. The inclusion of boys and gender-diverse participants, along with the refinement of response options, improved the instrument’s clarity and broadened its applicability for assessing gender-related disadvantages in diverse populations.

## Data Availability

The database that originated the article is available in an open repository (https://doi.org/10.25824/redu/BDDNEI).
